# Mechanical Characteristics of Sandwich Structures with 3D-Printed Bio-Inspired Gyroid Structure Core and Carbon Fiber-Reinforced Polymer Laminate Face-Sheet

**DOI:** 10.3390/polym16121698

**Published:** 2024-06-14

**Authors:** Harri Junaedi, Marwa A. Abd El-baky, Mahmoud M. Awd Allah, Tamer A. Sebaey

**Affiliations:** 1 Engineering Management Department, College of Engineering, Prince Sultan University, Riyadh 12435, Saudi Arabia; tsebaey@psu.edu.sa; 2 Mechanical Design and Production Department, Faculty of Engineering, Zagazig University, Zagazig 44519, Sharkia, Egypt; dr.marwa2013@yahoo.com (M.A.A.E.-b.); mmsawdallah@gmail.com (M.M.A.A.); 3 Industrial Department, College of Engineering, King Khalid University, Abha 61421, Saudi Arabia; 4 Mechanical Engineering Department, Southern Methodist University, Dallas, TX 75205, USA

**Keywords:** sandwich, bio-inspired, gyroid structure, 3D printing, PLA, CFRP, flexural test, mechanical properties, Weibull analysis

## Abstract

The gyroid structure is a bio-inspired structure that was discovered in butterfly wings. The geometric design of the gyroid structure in butterfly wings offers a unique combination of strength and flexibility. This study investigated sandwich panels consisting of a 3D-printed gyroid structure core and carbon fiber-reinforced polymer (CFRP) facing skin. A filament fused fabrication 3D printer machine was used to print the gyroid cores with three different relative densities, namely 10%, 15%, and 20%. Polylactic acid (PLA) was used as the printing material for the gyroid. The gyroid structure was then sandwiched and joined by an epoxy resin between CFRP laminates. Polyurethane foam (PUF) was filled into the gyroid core to fill the cavity on the core for another set of samples. Flexural and compression tests were performed on the samples to investigate the mechanical behavior of the sandwiches. Moreover, the two-parameter Weibull distribution was used to evaluate the results statistically. As a result, the sandwich-specific facing stress and core shear strength from the three-point bending test of the composites increased with the increase in sandwich density. Core density controls the flexural characteristics of the sandwich. Adding PUF improves the deflection at the maximum stress and the sustained load after fracture of the sandwich. Compression strength, modulus, and energy absorbed by gyroid core sandwiches and their specific properties are higher than the PUF-filled gyroid core sandwiches at equal sandwich density.

## 1. Introduction

The lightweight property of aircraft structures is essential. Lighter aircraft structure will improve fuel economy, payload capability, performance, and safety, leading to a reduction in CO_2_ emissions [1]. Sandwich structures are essential in the scope of aircraft design and construction due to their remarkable strength-to-weight ratio [2]. They are a type of composite comprised of two thin, high-strength outer layers frequently constructed from composite laminates or aluminum, with a lightweight core material, typically foam or honeycomb, sandwiched in between. This construction method provides benefits in the field of aircraft design, such as distributing and carrying the load effectively while providing stiffness and strength. Sandwich structures also have excellent vibration-damping properties, significantly reducing noise transmission and improving passenger comfort [3]. They also act as thermal insulators, maintaining the temperature in the interior [4].

Additive manufacturing (AM), or 3D printing, has transformed the manufacturing industry. Even though it has been recognized since the early 80s, it has grown in popularity in recent years as it has become increasingly affordable. Filament fused fabrication (FFF) is one of the most commonly used printing methods. The FFF method is a 3D printing method that requires an extrusion process of a continuous polymer filament in which the object is constructed by depositing layers of melted material [5]. Complex structures can be manufactured which enable new potential and novel applications with the existence of AM [6]. Although material selection is still challenging, it has altered the entire manufacturing industry’s perspective, opening new possibilities for designs that were previously unfeasible with conventional manufacturing methods. 

**Figure 1:**
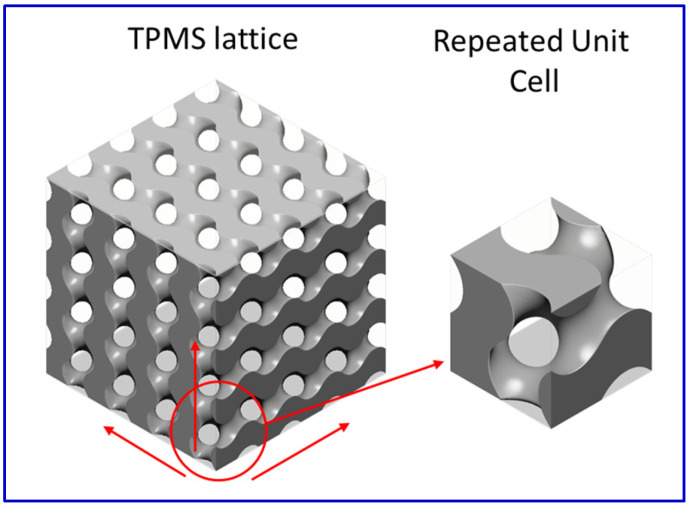
TPMS structure of gyroid structure. Arrows show the three directions of repeated unit cell.

The structured design inspired by nature has been considered a key mechanism for enhancing materials’ mechanical properties and functionality beyond their physical constraints [7]. Bio-inspired structures have become a trend in the structural industry [8,9]. Many novel materials have been developed that were inspired by nature. The gyroid is one of the bio-inspired structures that NASA scientist Alan Schoen discovered in 1970 [10]. It is a triply periodic minimal surfaces (TPMSs) structure that repeats itself in three different directions in space, like a crystal (Figure 1). Michielsen and Stavenga [11] discovered that the chitin in some butterfly wings is made to form a gyroid structure.

A gyroid is a complex structure that is impractical to construct using conventional manufacturing techniques. However, with 3D printing, this structure can be produced at a lower cost and in less time. Gyroid structures fabricated by AM and used as sandwich panels have been investigated by others [12,13,14]. Sandwich composites are widely used in aeronautics, buildings, and construction owing to their light weight and stiffness [15,16]. They employ high stiffness and strength material as facing skins and lightweight porous material as a core [17]. Pelanconi and Ortona [12] designed a lightweight sandwich panel with a gyroid structure core by AM at different thicknesses (0.375, 0.75, and 1.50 mm). Unidirectional carbon fiber reinforced ribs with different diameters (0.6, 1.2, and 2.4 mm) were applied on the face skins of the structure. They found that a structure with less gyroid thickness (0.375 mm) and a higher carbon fiber rib diameter (2.4 mm) has the optimum properties (density of 314 kg/m^3^ and stiffness of 25.8 GPa). Ayrilmis et al. [13] examined a sandwich panel of a gyroid core made via 3D printing by varying skin thickness (0.5, 1, 2, and 2.5 mm). The increase in skin thickness substantially enhances the mechanical properties. They recommend sandwich skins with a 2 mm or higher thickness for the sandwich panel.

Various combinations of facing sheets and cores of sandwich structures have been studied. Flexural, compression, and impact are the most common mechanical tests to evaluate the sandwiches. Different shapes of 3D-printed and bio-inspired core structures have been studied, such as primitive, honeycomb, rhombus, corrugated, diamond, and gyroid. Sugiyama et al. [18] investigated sandwich structures of different cores, e.g., honeycombs, rhombuses, rectangles, and circles, using a continuous carbon fiber 3D printer. They concluded that the sandwiches’ flexural strength and stiffness depend on the core’s density and shape. Zaharia et al. [19] studied blending polylactic acid and polyhydroxyalkanoate materials with a 3D printer to fabricate sandwich composites. Three geometry variations, namely honeycomb, diamond-celled, and corrugated, were used as cores to create unity with the skins. The main failures from the flexural test were reported as skin yielding, skin wrinkling, and core and skin debonding for diamond-cell, honeycomb, and corrugated cores, respectively. The highest flexural strength was shown by the diamond-celled core sandwich, reaching 16 MPa, which is two and three times higher than the honeycomb and corrugated structures.

Haldar et al. [20] exploited corrugated triangular and trapezoidal structures as the core material of sandwich composites. They investigated the effects of core thickness and height, skin thickness, and core-skin interface area on the mechanical characteristics of the sandwich. Their experimental results indicate that increased core thickness enhanced the sandwich panel’s compression strength and energy absorption. The core and the skin interface bonding area contribute significantly to the mechanical characteristics of the sandwich. Peng et al. [21] studied sandwich structure with TPMS cores of different geometries (primitive, neovius, and I-graph-wrapped package (IWP)), core densities, and face sheet thicknesses. The core density and geometric shapes significantly influenced the sandwiches’ flexural and energy absorption properties. Meanwhile, the core morphologies have a minimum impact on the flexural properties at lower core density for a thick facing skin.

A sandwich composite made of a bio-inspired 3D-printed gyroid structure core combined with a carbon-fiber-reinforced-polymer (CFRP) laminate facing sheet has been the subject of interest in this research. The purpose of this research is to study the mechanical characteristics of sandwich composites with different densities of gyroid structure core. Another investigated aspect was the influence of incorporating PUF material into the gyroid core. The gyroid core structures were manufactured via 3D printing using PLA filament material. Meanwhile, the facing sheet was made of CFRP laminate. The flexural and compression tests were performed on the sandwich composites. Different flexural properties of sandwich panels, such as maximum load, deflection before failure, facing stress, and core shear strength, were determined and compared. Compression strength, modulus, and energy absorbed up to 50% compression strain were also calculated. The failure progression from the flexural test of each configuration was also observed. Additionally, the experimental responses were statistically assessed using two parameters from the Weibull distribution.

## 2. Materials and Methods

### 2.1. Materials

Three-dimensional printing polylactic acid (PLA) filament, 2.85 mm in diameter with a density of 1.24 g/cm^3^, purchased from RlfdPlastics Co. (Riyadh, KSA, Saudi Arabia), was used in this research. The PLA, as a printed sample (0° raster angle to the axial direction), has a tensile strength of 50 MPa and an elastic modulus of 3500 MPa. Dragonplate, ALLRed & Associates Inc. (Elbridge, NY, USA) supplied carbon fiber-reinforced epoxy laminate (CFRP). The CFRP laminate had a thickness of 0.85 mm, a fiber volume fraction of 52%, and a 1.42 g/cm^3^ density. It consisted of 3 layers of carbon fiber (CF) fabric with plain and 2 × 2 twill woven fabric (0/90°) orientation on each side of the outer layers and 2 × 2 twill woven spread-tow (±45°) orientation on the center layer. The epoxy resin used as the matrix was 304-1 (Mitsubishi Chemical Carbon Fiber and Composites, Sacramento, CA, USA). The epoxy has a tensile strength of 57 MPa and an elastic modulus of 2900 MPa. The CF was Toray T300 (Toray Composite Materials America, Inc, Tacoma, WA, USA), with a tensile strength of 3530 MPa and a modulus of 230 GPa. The CFRP has a tensile strength of 450 MPa and an elastic modulus of 30 GPa. Meanwhile, the lap shear strength between CFRP and PLA is 3 MPa.

PLA was chosen due to its good printability, accuracy, relatively high strength, and elastic modulus compared to other 3D printing filaments, such as acrylonitrile butadiene styrene (ABS), polyethylene terephthalate glycol (PETG), and polypropylene (PP). CFRP was selected for its high strength and low weight. Meanwhile, PUF was chosen due to its low weight and easy insertion between the lattice of the gyroid core in liquid form. 

The PLA gyroid structure cores and CFRP skins were joined by epoxy resin. The foam material used to fill the sandwiches was rigid closed-cell Polyurethane foam (PUF) supplied by Fiberglass Warehouse (San Diego, CA, USA). It was a 1:1 by-weight mix ratio of two-component resin. The first component consists of polymethylene polyphenyl isocyanate and the second component is a mixture of diethylene glycol and polyether polyol as the main constituents and foaming agent of hydrofluorocarbon 1,1,1,3,3-pentafluoropropane (HFC-245FA). The density of the cured foam was about 32 kg/m^3^ (2 lb/ft^3^).

### 2.2. Specimen Fabrication

**Figure 2:**
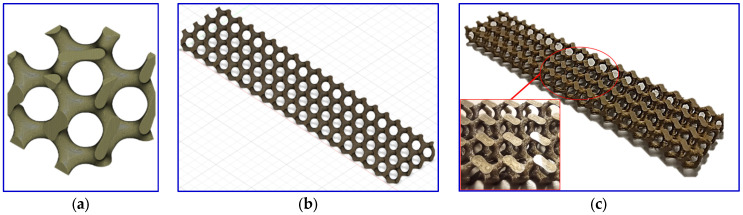
(**a**) Gyroid cubic shape unit cell, (**b**) design of gyroid structure, and (**c**) printed gyroid structure at 20% density.

Computer-aided design (CAD) software (Fusion 360 2.0.18961) with volumetric lattice extension was used to design gyroid structures with different relative densities, namely 10, 15, and 20%. The difference in densities resulted in varying the gyroid’s lattice strut thickness while maintaining the overall dimensions. The designed gyroid’s outer dimensions were 150 × 30 × 10 mm^3^ with a unit cell dimension of 8 mm^3^. The derived lattice strut thicknesses were around 1.4, 1.7, and 2 mm, respectively, for 10, 15, and 20% gyroid structures. Figure 2a,b show the gyroid cubic shape unit cell and the design of the gyroid structure, respectively. Then, the designs were converted into stereolithography (STL) extension files to be processed by 3D printer slicer software (Ultimaker Cura 5.2.1). All the parameters utilized were determined using the slicer software. The printing nozzle and build plate temperatures were set to 220 °C and 70 °C, respectively. The layer thickness, printing speed, and cooling fan speed were 0.1 mm, 70 mm/min, and 255 rpm, respectively. The printing pattern was concentric, and the density of the design was 100%. The 3D printer Ultimaker S5 (Zaltbommel, The Netherlands) was employed to print the gyroid structures. Figure 2c displays the printed gyroid structure at 20% of density as a representative sample.

The gyroid structure was then sandwiched between the CFRP laminates. Prior to joining, the meeting surfaces of the CFRP and gyroid structure were sanded with sandpaper to create roughness on the surface and promote bonding. The sanded surfaces were cleaned with isopropyl alcohol (IPA) and dried. A thin layer of epoxy resin was applied to the CFRP surface as an adhesive. Then, the gyroid PLA core was sandwiched between two CFRP laminates and clamped for 24 h. For another set of samples of filled PUF gyroids, the cavities of the gyroid core sandwiches with the same density were filled with PUF. Table 1 presents the different configurations of the sandwich samples. Sandwich density was then calculated by dividing the weight by the volume of each sandwich. 

### 2.3. The Flexural and Compression Tests

**Figure 3:**
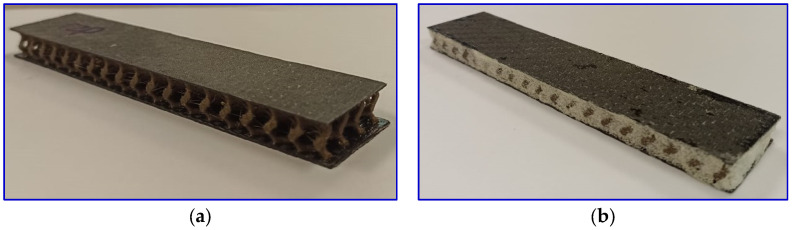
(**a**) Gyroid sandwich and (**b**) PUF-filled gyroid sandwich at 20% of gyroid core density.

**Figure 4:**
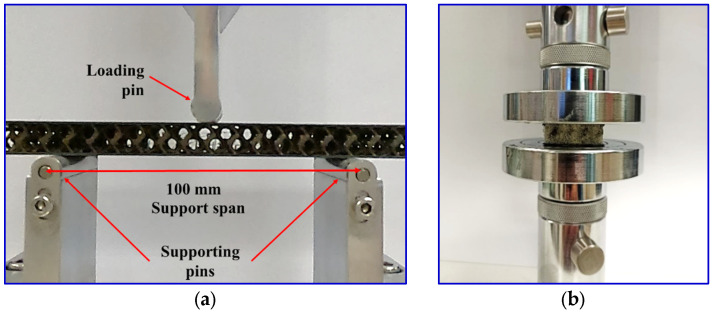
(**a**) Flexural test and (**b**) compression test of the sample setup.

A universal testing machine (WDW-10, Dongguan, China) was utilized to perform the flexural and compression tests. The flexural test was conducted using the three-point bending method according to the ASTM C393 standard [22]. The test was conducted at a crosshead speed of 2 mm/min until the sample failed. Three samples were assessed for every configuration. The samples’ overall dimensions are 150 × 30 × 11.7 mm^3^ (Figure 3). The distance between the supports of the sample was specified at 100 mm. The flexural test setup is displayed in Figure 4a. Ultimate core shear strength ($\sigma_{s}^{ult}$) and facing stress (*σ_f_*) at maximum load (*P_max_*) were determined corresponding to the ASTM standard C393 as displayed by Equations (1) and (2), respectively, as below:

(1) $$\sigma_{s}^{ult}=\frac{P_{max}}{\left(d+c\right)b}$$

(2) $$\sigma_{f}=\frac{P_{max}S}{2t(d+c)b}$$

where *b*, *c*, and *d* are the sample width, core thickness, and total sandwich thickness, respectively. Meanwhile, *S* is the three-point bending test support span, and *t* is the sandwich panel face-sheet thickness. All tests were subjected to video recording to identify the sandwiches’ failure mechanism.

A compression test was performed on the sandwich panel according to the ASTM standard C365 [23] with a 30 × 30 mm^2^ sample dimension and a 10 mm core thickness. Figure 4b displays the experiment setups for the compression test. A compression speed of 0.5 mm/min was employed. The samples were crushed up to 50% of the strain, and three repetitions were performed for each configuration. The strength, modulus, and energy absorbed by up to 50% strain of the sandwich composites were then determined. 

### 2.4. Statistical Analysis

Despite the fact that samples are produced and experimented in the same conditions, there is considerable variability in the experimental test results. As reported by Selmy et al. [24], Abd El-baky [25], and Awd Allah et al. [26], in assessing failure probability, the Weibull function proves to be a beneficial technique for characterizing the attributes of composite materials. A two-parameter Weibull distribution function can be performed to analyze the results statistically. The probability density function (f(n)) and the cumulative distribution function ($F\left(n\right)$) were utilized to analyze the experimental results, which are defined as follows [27,28]:

(3) $$f\left(n\right)=\left(\frac{\alpha }{\beta }\right)\left(\frac{n}{\beta }\right)exp\left[-\left(\frac{n}{\beta }\right)\right]$$

(4) $$F\left(n\right)=1-exp\left[-{\left(\frac{n}{\beta }\right)}^{\alpha }\right]$$

The α and β parameters of the two-parameter Weibull function correspond to the shape and scale parameters, respectively. Meanwhile, n is the variable value.

#### 2.4.1. Determining *α* and *β* Graphically

The reliability function $L_{R}\left(n\right)$ is specified as $L_{R}\left(n\right)=1-F(n)$. Substituting F(n) by Equation (4), $L_{R}\left(n\right)$ can be written as

(5) $$L_{R}\left(n\right)=exp\left[-{\left(\frac{n}{\beta }\right)}^{\alpha }\right]$$

Applying the natural logarithm to both sides of Equation (5) two times generates as follows

(6) $$\ln\left[\ln\left(\frac{1}{L_{R}\left(n\right)}\right)\right]=\alpha \ln\left(n\right)-\alpha \ln\left(\beta \right)$$

The relation between $\ln\left[\ln\left(\frac{1}{L_{R}\left(n\right)}\right)\right]$ and $\ln\left(n\right)$ in Equation (6) is linear, and α is the slope of the line. The β can be retrieved from the 2nd term of Equation (6). Firstly, the variable n was initially arranged in ascending order, and a sequential integer was assigned for the respective value (i=1, 2, 3, …,k). Then, the ${\;L}_{R}$ of each n was computed by the following formula:

(7) $$L_{R}=1-\left[\frac{\left(i-0.3\right)}{\left(k+0.4\right)}\right]$$

where k is the total number of the tested specimens.

#### 2.4.2. Scatter in Test Results

The mean (*M*), standard deviation (SD), and coefficient of variation (CV) of the experimental result values were determined by the below equations [29]:

(8) $$M=\beta \Gamma \left(1+\frac{1}{\alpha }\right)$$

(9) $$SD=\beta \sqrt{\left[\Gamma \left(1+\frac{2}{\alpha }\right)-\Gamma^{2}\left(1+\frac{1}{\alpha }\right)\right]}$$

(10) $$CV=\frac{SD}{Mean}=\frac{\sqrt{\left[\Gamma \left(1+\frac{2}{\alpha }\right)-\Gamma^{2}\left(1+\frac{1}{\alpha }\right)\right]}}{\Gamma \left(1+\frac{1}{\alpha }\right)}$$

where *Γ* is the gamma function. The *CV* characterizes the scatter in the data. The scatter reduces as the *CV* approaches zero.

## 3. Results

### 3.1. Flexural Test

**Figure 5:**
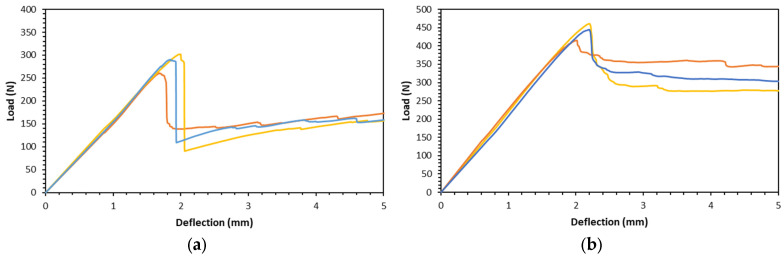
Repeatability of the flexural tests of (**a**) gyroid at 10% density (d10) and (**b**) PUF-filled gyroid at 10% density (d10P).

Figure 5 shows the load-deflection results for specific configurations from the flexural test to show the repeatability of the tests. It exhibits respectable reproducibility. The curves for the same configuration have a similar profile and character. The consistent behavior observed in the load-deflection curves also indicates that the sandwich being produced exhibits constant mechanical properties under the applied loads. This stability is essential in materials because it suggests that their performance can be predicted with a reasonable degree of accuracy. This is crucial for applications in industries where structural integrity and dependability are essential. 

**Figure 6:**
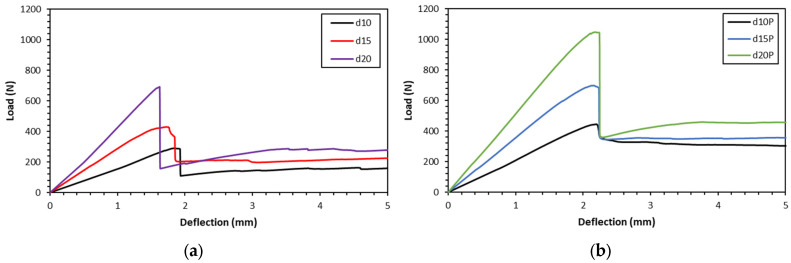
Representative force-deflection curves of the sandwiches from three-point bending (**a**) gyroid core sandwiches and (**b**) PUF-filled gyroid core sandwiches.

Figure 6 shows the load-deflection curve for the sandwiches. They have a similar type of curve: the load increases with the increase in deflection and then reaches a maximum, followed by a load drop to a certain level. Thereafter, the load tends to be maintained or slightly increased by the increase in deflection. For gyroid cores only, the load drops to around 100–200 N (see Figure 6a); meanwhile, for PUF-filled gyroid cores, the drop is to a higher level, which is around 350–400 N (see Figure 6b).

From the analysis of the load-deflection curve, important parameters such as the maximum load and the corresponding deflection at this point are determined, and their values are reported in Table 2. A visible trend appears upon analyzing the table, revealing that the maximum load experiences a proportional increase with the increasing sandwich density in both cases, the gyroid or the PUF-filled gyroid core. Incorporating PUF into the gyroid core enhances the maximum bending load capability while simultaneously raising the overall sandwich density. The flexural deflection at maximum load tends to decrease with the increase in gyroid core density; meanwhile, it tends to be constant for PUF-filled gyroid cores. 

From the load-deflection curves, facing stress and core shear strength were calculated, and the results are presented in Table 3. Adding PUF to the gyroid core enhanced the facing stress and core shear strength of the sandwiches. The facing stress and core shear strength of the sandwich panel tend to increase with the increase in sandwich density with and without PUF.

### 3.2. Compression Test

**Figure 7:**
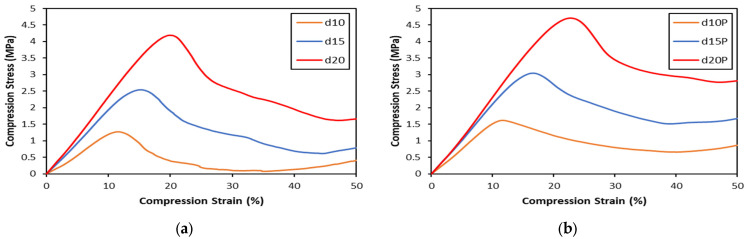
Compression stress–strain curves for (**a**) gyroid core sandwiches and (**b**) PUF-filled gyroid core sandwiches.

The compression stress–strain curves for the sandwich panels are shown in Figure 7, demonstrating that the profiles are constant across all configurations. The curves start with a linear slope within the elastic area, reaching a peak at the moment of the initial collapse of the core structure. This is followed by the stress gradually reducing with a plateau shape. Subsequently, an increase in stress occurs due to the structure’s densification. As soon as the collapsed wall cores come into contact with one another, densification takes place. The compression characteristics of the gyroid structure display a gradual decrease in stress after reaching the maximum stress. 

Compression strength, modulus, and energy absorbed from the compression test are displayed in Table 4. Compression strength, modulus, and energy absorbed increase with the gyroid core density for both unfilled and PUF-filled sandwiches. The addition of PUF increases the compression properties, which also increases the sandwich density (Table 2). 

## 4. Discussion

### 4.1. Flexural Properties

The sandwich composite with the addition of PUF has a higher deflection and force after initial failure, as can be seen in Figure 6. Adding PUF in the core can arrest the fracture of the gyroid core. The brittleness of the gyroid core, which is made of PLA material, is characteristic of the material. The brittleness of the PLA material in this sandwich composite would typically make the gyroid core more susceptible to catastrophic fractures and lead to structural failures. However, catastrophic failure on the gyroid core did not lead to the complete failure of the sandwich structure. The remaining intact core on the CFRP face sheet can still support a load. Moreover, the addition of PUF improved the ability of the sandwich structure to sustain a load after failure. PUF acts as a supporting structure that provides the composite structure with flexibility. The PUF addition results in a considerably higher value of deflection at maximum load. This implies that the addition of PUF enhances the structural capacity to withstand bending loads by improving the maximum load and deflection at maximum load.

The core density affects the facing stress and core shear strength of the sandwiches. The failure occurred on the gyroid lattice structure due to shear stress. The lattice topology of the gyroid structures with different densities was identical. The only difference was in the thickness of the lattice. The higher the density of the gyroid structure, the bigger the lattice thickness. A larger lattice thickness can support a higher shear load. Thus, the thickness of the lattice controls the properties of the sandwiches [30].

**Figure 8:**
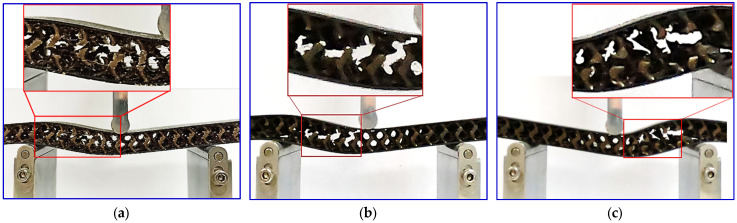
Fracture samples of (**a**) 10%, (**b**) 15%, and (**c**) 20% of gyroid core sandwiches.

The failure modes observation of each gyroid core sandwich are presented in Figure 8. It can be seen that core shear failure dominates the mode of failure in these gyroid core sandwiches. This observation indicates that the interfacial bonding between the core material and the face sheet is strong enough to withstand the shear forces on the interface experienced during the flexural test. The core shear failure typically initiates at the center of the core material, where the stress is concentrated. Following the initial failure of the gyroid lattice in this region, failures tend to propagate horizontally through the middle of the core material. There are no signs of failure or damage on the face sheet, which is made of a stronger material than the core. Facing stress developed on the facing skin is still much lower than the facing skin strength. No local indentations were discovered on the upper skin of the sandwich structure during the flexural test. This result is explained by the reasonable compression strength of the gyroid structures, which inhibit local indentation or bending of the top-facing sheet.

**Figure 9:**
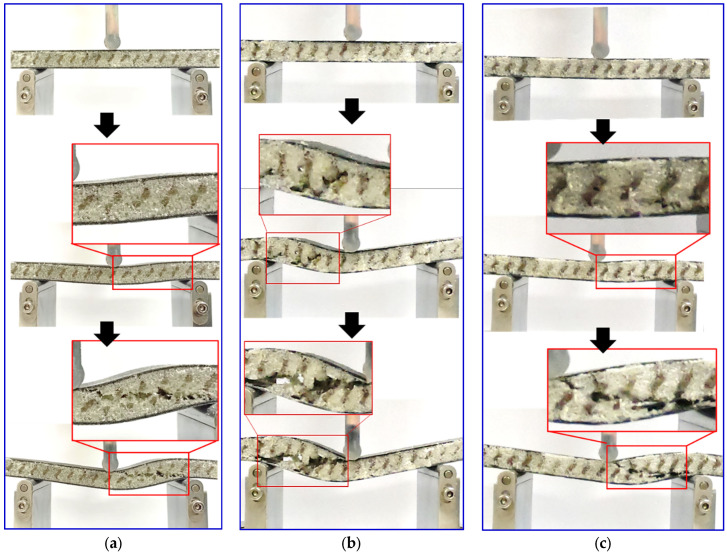
Fracture progression of (**a**) 10%, (**b**) 15%, and (**c**) 20% of PUF-filled gyroid core sandwiches.

Similar to the gyroid core, failure is initiated from the core of a PUF-filled gyroid core sandwich due to shear stress. What distinguishes this particular sandwich panel is the failure progression. To further explain these observations, Figure 9 presents a visual representation analysis of the panel’s behavior under the bending load. The PUF-filled gyroid sandwich panel failure occurred across the top skin to the bottom skin. Then, it propagated on the interface between the core and skin, as shown in Figure 8, which is the typical core shear failure of foam core sandwich composite [31,32,33]. The presence of PUF controls and restrains the fracture of the gyroid lattice. This suggests that the addition of PUF enhances the core’s structural properties and improves the sandwich panel’s overall bending performance.

### 4.2. Compression Properties

**Figure 10:**
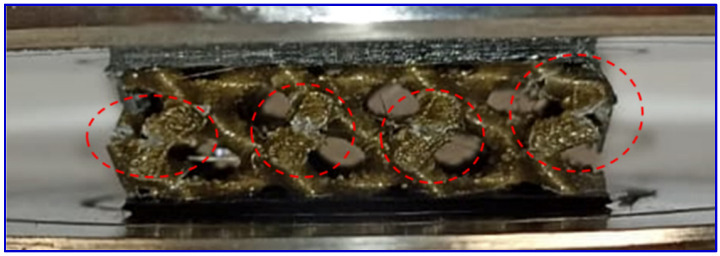
Compression sample of 20% relative density gyroid core sandwich during test.

A gradual decrease in the compression stress–strain curve was observed in Figure 6. This gradual decrease is an indication of an instability elastic buckling or plastic yielding on the compression sample failure [34]. Figure 10 shows the failure of the gyroid structure due to compression load. This figure shows a lattice strut buckling of the outer struts of the structure followed by fracture (show by red dash circle). Since the structure was fixed to the top and bottom facing skins, the stretch of the core in the lateral direction is limited, resulting in an elastic instability buckling followed by a fracture on the lattice strut of the gyroid structure in the middle. Multiple fractures from buckling on the lattice struts were observed in all the samples. Peng et al. [35] reported that plastic yielding and elastic buckling may occur in a gyroid structure, depending on the slenderness ratio. Such structures have potential for energy absorption applications compared to structures prone to sudden or catastrophic failure.

### 4.3. Specific Mechanical Properties

**Figure 11:**
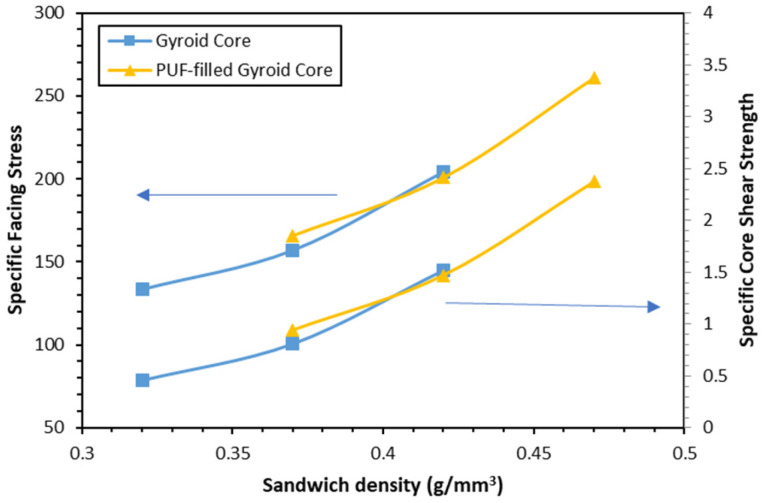
Facing stress and core shear strength vs. density comparison.

Table 5 exhibits the specific mechanical characteristics of the designed structures. Specific properties were calculated by normalizing the mechanical properties by the sandwich density. The addition of PUF to the gyroid core has improved the specific facing stress and core shear strength. The PUF-filled gyroid core sandwiched has similar strength compared to only the gyroid at the same sandwich density. It is clear from Figure 11 that the facing stress and core shear strength properties of the gyroid and PUF-filled gyroid sandwich panels show exponential increases in facing stress and core shear strength with increasing sandwich density. The figure demonstrates the sandwich’s face stress and core shear strength of gyroid and PUF-filled gyroid curves with the same density overlap. In conclusion, these two properties depend highly on the sandwich density for this case.

Furthermore, the specific compression strength and modulus for the gyroid core and PUF-filled gyroid core sandwiches were compared. There is a noticeable improvement in specific compression strength as the sandwich density increases. This density-dependent performance emphasizes core density’s role in the compression properties of overall sandwich structures. Additionally, they exhibit higher specific compression strength and modulus when comparing gyroid core and PUF-filled sandwiches at the same sandwich densities. This suggests the gyroid core contributes more to mechanical performance in compression than the PUF. 

In addition, it is seen that the specific compression modulus is not significantly affected by the addition of PUF, especially at 15% and 20% of the gyroid relative density. This result emphasizes that the gyroid core primarily regulates the compression modulus. Furthermore, the practical implications of these mechanical characteristics become apparent in flexural evaluations, wherein the compression strength of the gyroid core is considered adequate to prevent localized bending or indentation on the sandwich’s top-facing surface beneath the loading pin. This observation implies that the compression strength of the gyroid core effectively preserves the sandwich panel’s structural integrity, thereby mitigating potential problems associated with low core density, skin strength, and stiffness [36,37]. Such issues may otherwise result in localized bending or indentation when subjected to loading conditions.

The specific energy absorbed during the compression tests of both types of sandwiches is presented. The energy absorbed demonstrates a linear enhancement with the progressive increase in sandwich density. This relationship underlines the critical role that sandwich or core density plays in influencing the capacity of the sandwiches to absorb energy under compression loading conditions. Furthermore, a comparative analysis between the two types of sandwiches indicates that, at equivalent sandwich densities, the specific energy absorbed by the unfilled sandwich is higher than that of the PUF-filled gyroid core sandwich. This observation aligns with the earlier-discussed trend in compression strength, where the unfilled variant exhibited higher compression strength. The unfilled sandwich composite has superior energy absorption compared to the PUF-filled sandwich. It implies that the gyroid core structure and material have higher energy absorption than the PUF structure at the same density. The combination of core densities, topologies, and materials determines the sandwich composite’s specific properties, which can be tailored to meet specific requirements.

### 4.4. Statistical Analysis

**Figure 12:**
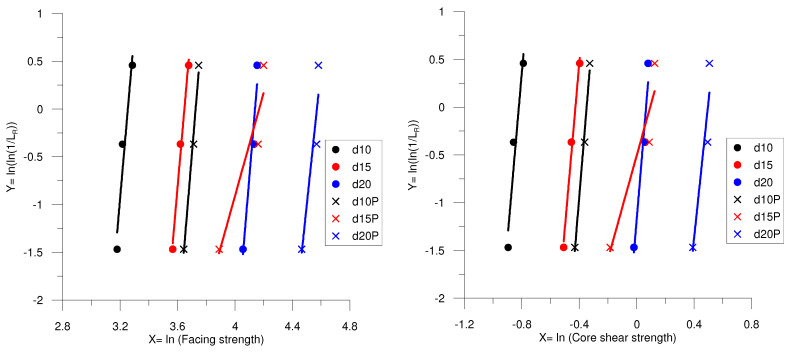

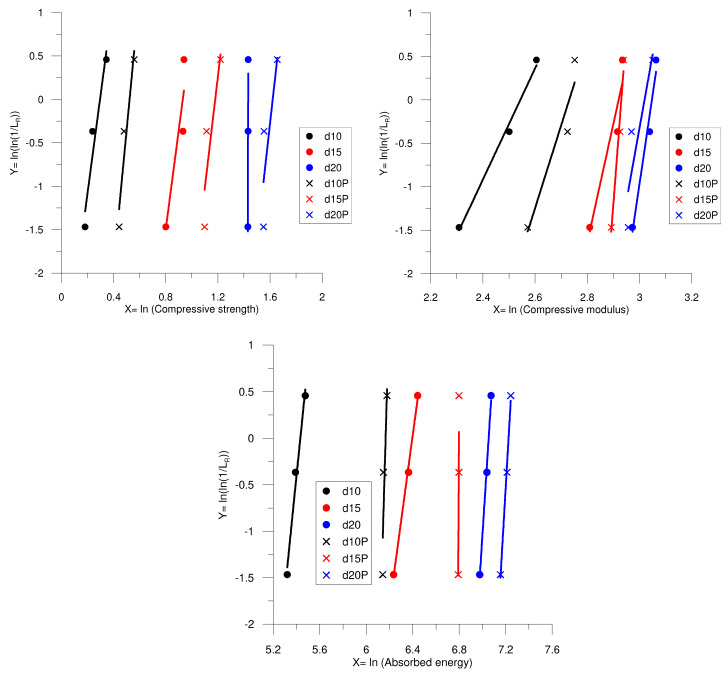
Graphical analysis of the flexural and compression properties.

Figure 12 depicts the graphical analysis used to determine the parameters (α) and (β) of the two-parameter Weibull distribution functions for the obtained data. It should also be noted that the points highlighted in Figure 11 correspond to the actual data distribution acquired from the experiments for every sandwich panel. Table 3 and Table 4 display the test results M, SD, and CV for the flexural and compression properties, respectively. The maximum CV was reported to be 21.32% in d15P for both the facing stress and core shear strength. However, d10 recorded the maximum CV for compression strength and compression modulus with values of 10.728 and 18.284%, respectively. Additionally, d15 noted the CV for energy absorbed with a value of 12.521%. All of the CV values are smaller than 22%, indicating an obvious accuracy level for all of the designed structures tested. According to Awd Allah et al. [38], the scatter in the results is due to manufacturing flaws.

## 5. Conclusions

Bio-inspired gyroid structures were investigated at varying densities as the core material of sandwich panels. In addition, the effects of adding PUF material to the core were studied, in combination with the application of gyroid core structures. These gyroid core structures were sandwiched between CFRP laminates to create composite sandwich panels. The study aimed to understand how different parameters, such as core density and topology, influence the flexural properties of these sandwich panels. As the density of the core structure increased, the sandwiches exhibited a significant increase in the maximum bending load, facing stress, and core shear strength. Two parameters from the Weibull distribution were used to assess the experimental results statistically. The exponential relationship between density and these attributes suggests that higher density and thicker lattice structures considerably improved sandwich performance. When comparing sandwich panels with a gyroid structure core to those with a PUF-filled gyroid core, it was found that, at the same sandwich density, the facing stress and core shear strength were comparable. This result implies that the density of the sandwich had a more significant impact on the flexural properties than the topology of the core material. Combining the gyroid structure with PUF within the core had a notable advantage, improving the flexural deflection of the sandwich panels before reaching the point of failure. This attribute is beneficial since the sandwich panels exhibit high strength and toughness. In terms of failure behavior, core shear failure was identified as the only initial failure mode in both sandwich panels. Compression strength, modulus, and energy absorbed increase with sandwich density for both cases. Gyroid core sandwich compression properties are higher compared to PUF-filled gyroid sandwiches at the same level of sandwich density. Based on the statistical analysis, all the designed structures put to the test replicate a noticeable level of accuracy. The combination of bio-inspired gyroid structures and PUF shows potential in achieving strong composite materials with various possible applications for industries such as aircraft and automotive. An area for future work could involve comparing different topologies of 3D-printed cores to compare and optimize their mechanical properties.

## Figures and Tables

**Table 1:** Configuration of the sandwich panels.

Name Tag	Designed Relative Density of Gyroid Core (%)	Bulk Density of Gyroid Core (g/cm^3^)	PUF Filled
d10	10	0.13	No
d15	15	0.19	No
d20	20	0.25	No
d10P	10	0.13	Yes
d15P	15	0.19	Yes
d20P	20	0.25	Yes

**Table 2:** Sandwich density, maximum load, and deflection before failure of the sandwich panel from the flexural test.

Name Tag	Sandwich Density (g/mm^3^)	Max. Load (N)	Deflection at Max. Load (mm)
d10	0.32 ± 0.01	282 ± 19	1.96 ± 0.25
d15	0.37 ± 0.01	418 ± 30	1.5 ± 0.21
d20	0.42 ± 0.01	681 ± 42	1.65 ± 0.17
d10P	0.37 ± 0.01	441 ± 23	2.14 ± 0.11
d15P	0.42 ± 0.01	651 ± 105	1.97 ± 0.3
d20P	0.47 ± 0.01	1018 ± 64	2.16 ± 0.11

**Table 3:** Facing stress and core shear strength of the sandwiches.

Name Tag	Data	Facing Stress (MPa)	Core Shear Strength (MPa)
d10	α	17.34	17.34
	β	25.89	0.44
	(*M*)	25.11	0.43
	(*SD*)	1.79	0.03
	(*CV*) %	7.11	7.114
	Eqn.	Y = 17.34 X − 56.42	Y = 17.340 X + 14.23
d15	α	17.23	17.23
	β	38.40	0.65
	(*M*)	37.23	0.63
	(*SD*)	2.67	0.05
	(*CV*) %	7.16	7.16
	Eqn.	Y = 17.23 X − 62.84	Y = 17.23 X + 7.35
d20	α	18.08	18.08
	β	62.80	1.07
	(*M*)	60.98	1.04
	(*SD*)	4.17	0.07
	(*CV*) %	6.84	6.83
	Eqn.	Y = 18.08 X − 74.84	Y = 18.08 X − 1.18
d10P	α	18.24	18.24
	β	41.52	0.71
	(*M*)	40.33	0.69
	(*SD*)	2.73	0.05
	(*CV*) %	6.77	6.77
	Eqn.	Y = 18.24 X − 67.98	Y = 18.24 X + 6.36
d15P	α	5.41	5.41
	β	64.64	1.10
	(*M*)	59.61	1.01
	(*SD*)	12.71	0.22
	(*CV*) %	21.32	21.32
	Eqn.	Y = 5.41 X − 22.54	Y = 5.41 X − 0.51
d20P	α	14.27	14.27
	β	96.62	1.64
	(*M*)	93.15	1.58
	(*SD*)	7.99	0.14
	(*CV*) %	8.58	8.58
	Eqn.	Y = 14.27 X − 65.22	Y = 14.27 X − 7.08

**Table 4:** Mechanical properties of sandwiches from a compression test.

Name Tag	Data	Compression Strength (MPa)	Compression Modulus (kPa)	Energy Absorbed (kJ/m^3^)
d10	α	11.28	6.39	12.31
	β	1.35	12.73	228.82
	(*M*)	1.29	11.85	219.48
	(*SD*)	0.14	2.17	21.70
	(*CV*) %	10.73	18.28	9.89
	Eqn.	Y = 11.28 X − 3.35	Y = 6.39 X − 16.25	Y = 12.31 X − 66.86
d15	α	11.45	13.66	9.24
	β	2.54	18.55	599.77
	(*M*)	2.43	17.86	568.63
	(*SD*)	0.26	1.60	71.20
	(*CV*) %	10.58	8.94	12.52
	Eqn.	Y = 11.45 X − 10.66	Y = 13.66 X − 39.89	Y = 9.24 X − 59.12
d20	α	634.34	20.40	19.54
	β	4.19	21.07	1156.82
	(*M*)	4.18	20.52	1125.51
	(*SD*)	0.001	1.25	71.31
	(*CV*) %	0.02	6.08	6.34
	Eqn.	Y = 634.34 X − 908.52	Y = 20.40 X − 62.16	Y = 19.54 X − 137.80
d10P	α	15.73	9.51	43.53
	β	1.69	15.35	476.58
	(*M*)	1.63	14.57	470.50
	(*SD*)	0.13	1.84	13.65
	(*CV*) %	7.81	12.62	2.90
	Eqn.	Y = 15.73 X − 8.23	Y = 9.51 X − 25.97	Y = 43.53 X − 268.41
d15P	α	12.82	39.41	267.97
	β	3.25	18.74	895.53
	(*M*)	3.13	18.47	893.61
	(*SD*)	0.30	0.59	4.01
	(*CV*) %	9.51	3.19	0.45
	Eqn.	Y = 12.82 X − 15.12	Y= 39.41 X − 115.50	Y = 267.97 X − 1821.49
d20P	α	13.50	16.80	21.51
	β	5.06	20.48	1373.26
	(*M*)	4.87	19.85	1339.22
	(*SD*)	0.40	1.46	77.32
	(*CV*) %	9.04	7.33	5.77
	Eqn.	Y = 13.50 X − 21.88	Y = 16.80 X − 50.72	Y = 21.51 X − 155.37

**Table 5:** Specific properties of the designed structures.

Name Tag	Flexural Test		Compression Test		
	Specific Facing Stress (MPa.mm^3^/g)	Specific Core Shear Stress (MPa.mm^3^/g)	Specific Compression Strength (MPa.mm^3^/g)	Specific Compression Modulus (kPa.mm^3^/g)	Specific Energy Absorbed (kJ.mm^3^/g)
d10	78.47	1.33	4.02	37.02	685.86
d15	100.63	1.71	6.56	48.26	1536.85
d20	145.18	2.47	9.96	48.86	2679.78
d10P	109.00	1.85	4.41	39.38	1271.62
d15P	141.94	2.41	7.44	43.98	2127.63
d20P	198.20	3.37	10.35	42.23	2849.39

## Data Availability

The original contributions presented in the study are included in the article. Further inquiries can be directed to the corresponding authors.
